# Incidence, Prevalence, Risk Factors, and Clinical Treatment for Children with Developmental Dysplasia of the Hip in Saudi Arabia. A Systematic Review

**DOI:** 10.1007/s44197-024-00217-5

**Published:** 2024-03-14

**Authors:** Naif Alrashdi, Mansour Alotaibi, Moqfa Alharthi, Faizan Kashoo, Sultan Alanazi, Ahmad Alanazi, Msaad Alzhrani, Thamer Alhussainan, Rami Alanazi, Rakan Almutairi, Matthew Ithurburn

**Affiliations:** 1 Department of Physical Therapy and Health Rehabilitation, College of Applied Medical Sciences, Majmaah University, AL-Majmaah, 11952 Saudi Arabia; 2 Department of Physical Therapy, College of Applied Medical Sciences, Northern Border University, Arar, Saudi Arabia; 3 Rehabilitation Services Department, King Abdullah Specialized Children’s Hospital, Ministry of National Guard Health Affairs, Riyadh, Saudi Arabia; 4 Department of Orthopedics, King Faisal Specialist Hospital & Research Center, Riyadh, Saudi Arabia; 5 Department of Physical Therapy and Rehabilitation, King Khaled Hospital, Almajmaah, Saudi Arabia; 6 Physiotherapy Department, Al Iman General Hospital, Riyadh First Health Cluster, Riyadh, Saudi Arabia; 7 American Sports Medicine Institute, Birmingham, AL USA; 8 Department of Physical Therapy, School of Health Professions, University of Alabama at Birmingham, Birmingham, AL USA

**Keywords:** Disability, Early screening, Acetabular dysplasia, DDH, Infants, Saudi arabia

## Abstract

**Background:**

Developmental dysplasia of the hip (DDH) leads to pain, joint instability, and early degenerative joint disease. Incidence, prevalence, and management strategies of DDH have been well-documented in several countries, but not in Saudi Arabia.

**Objective:**

We synthesized the current evidence regarding incidence, prevalence, risk factors, and clinical treatment for children with DDH in Saudi Arabia.

**Methods:**

We searched 3 databases to locate studies. Studies that included children with DDH in Saudi Arabia; reported either incidence rate, prevalence, risk factors, and/or clinical practice; and were available in English or Arabic were included. We excluded reviews, case studies, or animal studies. Two independent authors reviewed potential studies and assessed study’s quality.

**Results:**

Our search yielded 67 potential studies, of which 16 studies were included (total DDH sample = 3,127; age range = 2.5 to 86.4 months). Three studies reported incidence rates ranging from 3.1 to 4.9 per 1000 births, and 3 studies reported prevalence ranging from 6 to 78%. Nine studies reported that female sex, breech position, family history, and age less than 3 years were risk factors associated with DDH. Four studies reported that brace applications and closed reduction were conservative treatments, and 9 studies reported that open hip reduction, adductor tenotomy, and/or pelvic osteotomy were surgical approaches to treat DDH.

**Conclusions:**

In Saudi Arabia, the Incidence and prevalence rates of DDH are 3.1 to 4.9 per 1,000 births, and 6–78%, respectively **(**differ from what has been reported in other countries**)**, but the risk factors of DDH in Saudi Arabia appear to be similar in comparison to other countries (female, breech presentation, family history of DDH).

**Supplementary Information:**

The online version contains supplementary material available at 10.1007/s44197-024-00217-5.

## Introduction

Developmental dysplasia of the hip (DDH) is defined as incomplete bony growth of the hip joint that leads to incongruency between the femoral head and the acetabulum [1]. DDH varies in severity, from mild to severe, and is classified as either acetabular dysplasia, femoral head subluxation, or femoral head dislocation [2]. Several risk factors have been associated with DDH development in the literature, including improper swaddling, consanguineous marriage [3], breech presentation, female sex, positive family history (genetic predisposition), firstborn status, and oligohydramnios [4–6]. The global incidence rate of DDH varies significantly based on race, ethnicity and/or country [7, 8]. Specifically, previous studies have shown higher rates among Hispanic individuals and lower rates among Black populations [7]. According to DDH distribution across various countries, Zimbabwe has the lowest reported incidence rates of DDH cases (0.06 per 1000 infants) [9]. The American Academy of Pediatrics reported that the incidence of DDH among children was 11.5 per 1000 live births; 4.1 per 1000 for boys, and 19 per 1000 for girls [10]. The annual prevalence of DDH in the United States has been estimated as 1.7 per 1000 infants [11]. Understanding the epidemiology of DDH aids in targeted prevention through early screening programs, reducing disability and morbidity rates [12].

In DDH screening, early hip joint testing, using either clinical examination or imaging [13], is crucial, as normal locomotion in children with DDH is contingent upon early diagnosis and treatment [14]. Experts have advocated for the implementation of early DDH screening [15] and have emphasized the need to initiate interventions within the first month of life [15]. Most commonly-used methods for detecting DDH in neonates include clinical screening with the Ortolani maneuver (for hip dislocation) [16] and the Barlow test (for hip subluxation) [17]. Ultrasound imaging can be a more advanced screening option for individuals at increased risk [18]. Graf and colleagues first proposed the use of ultrasonography for the detection of DDH cases in the 1980s [19]. Since then, many ultrasound screening techniques have emerged [20, 21], which can be categorized into two broad categories: static tests that evaluate morphology and dynamic tests that evaluate hip joint stability. Previous work has shown that, at 4.5 months of age, when the femoral head is predominantly cartilaginous, ultrasound is most effective [22]. In addition, conventional anteroposterior pelvic radiographs are more beneficial [23].

Early DDH treatment lead to optimal alignment between the acetabulum and femoral head and allow for the hip joint to continue developing and growing normally [24]. Of concern, a delayed diagnosis may require more invasive treatment, such as surgery, that may be associated with complications, leading to both poor functional outcomes and quality of life [25]. Children with DDH that is identified early can be treated using conservative approaches, including static or dynamic brace applications, such as the Pavlik harness (dynamic splint) [26–28]. If conservative treatments are unsuccessful, then surgical intervention may be recommended, including open reduction techniques, which may involve performing a femoral or pelvic osteotomy [29]. Importantly, surgical interventions for DDH cases are associated with specific complications such as avascular necrosis of the femoral head, sciatic nerve injury, and/or femoral fracture [29].

In Saudi Arabia, one previous study reported that incidence rate of DDH was approximately 3.5 per 1000 births [30]. Other previous published studies have only focused on investigating the incidence, prevalence, risk factors, and/or clinical practice of DDH in Saudi Arabia within specific institutions or geographical regions [31–39]. To the best of our knowledge, no published studies have synthesized evidence regarding incidence, prevalence, risk factors, and/or clinical practice in children with DDH in Saudi Arabia. Therefore, pooling all information regarding epidemiological data related to DDH holds considerable appeal to inform future preventative healthcare strategies that aim to mitigating disability, morbidity, and economic burden associated with DDH condition, specific to the Saudi Arabian population. In this systematic review, we determined the incidence, prevalence, risk factors, and clinical approaches used among children with DDH in Saudi Arabia using the currently-available evidence. We hypothesized that the incidence and prevalence would differ, but not risk factors and clinical approaches, in children with DDH in Saudi Arabia compared to data reported from other countries and ethnicities.

## Methods

In this systematic review, we developed a comprehensive, detailed protocol (shared amongst team members) which outlined the key terms and search strategy, relevant databases, and screening processes for potential studies as well as data extraction and data synthesis from included studies. Our protocol was registered in the International prospective register of systematic reviews (PROSPERO: CRD42023433646). We followed the Preferred Reporting Items for Systematic Reviews and Meta-Analyses (PRISMA) Guidelines [40] for conducting and reporting this systematic review.

### Study Searches and Selection

Our inclusion criteria involved studies that: (1) included children diagnosed with DDH from all ages in Saudi Arabia, (2) reported one or more of the following: incidence rate, prevalence, risk factors associated with DDH, and/or clinical practice related to DDH, and (3) were available in full-text in either English or Arabic. We excluded reviews, conference proceedings or abstracts, case studies (*n* = 1), studies included children with other physical disorders and animal studies. We searched PubMed, Scopus, and CINAHL databases on June 1st, 2023, from inception, using relevant key terms. The key terms included synonyms and controlled vocabularies for DDH, incidence rate, prevalence, and clinical practice. Table 1 shows our search strategy and search terms. In addition to searching the electronic databases, we searched the reference lists of included studies to further locate other potential studies that did not appear in the database search. Two authors (NA, MA) independently screened titles and abstracts to determine initial potential eligibility and then full-text review. We used Covidence (Veritas Health Innovation, Melbourne, Australia) to maintain consistency, track screening, and generate PRISMA flowcharts. In the case of discrepancy between the two authors (NA, MA) regarding included studies, a third author (MA) determined the final decision of inclusion or exclusion [41].

**Table 1:** Search strategy (PubMed example)

Database searched	Date of Search	Search Terms
PubMed	June 1st 2023 (searched from inception)	1.((“Developmental dysplasia of the hip “[Mesh] OR “DDH“[Mesh] OR “Congenital hip dislocation “[Mesh] OR " Congenital subluxation of the hip " [Mesh] OR " Congenital dysplasia of the hip”)[Mesh] AND (“Saudi Arabia” [Mesh]) AND (“incidence” OR “prevalence” OR “Risk Factors” OR “Medical Management” OR “Surgical Management”) NOT (“Review” [Publication Type] OR review OR “Systematic Review” [Publication Type]))

### Data Extraction

All authors met and agreed on the main outcomes/measures that would be extracted from the included studies. One author (NA) drafted the data extraction sheet, and that sheet was shared among all authors. The authors provided feedback on items included in that data extraction sheet. After considering feedback from the authorship team, we reached a final data extraction sheet. Two authors (NA, MA) independently used the sheet to extract relevant data from the included studies. The two authors met and discussed the extracted data and resolved any conflicts or missing information between the two, independent data extraction sheets. After resolving conflicts and missing information, we finalized a complete consensus data extraction sheet and used these data to synthesize findings for the present systematic review. Study-related demographic, and clinical data extracted included the first author’s last name and year of publication, sample size, average age, sex distribution, involved side, DDH diagnostic criteria used, and associated anomalies with DDH. We also extracted the main outcomes/measures of interest including incidence, prevalence, risk factors, and/or clinical practice approaches for children with DDH in Saudi Arabia.

In this systematic review, we defined incidence rate as any reported value (ratio) for newly diagnosed DDH cases in Saudi Arabia, and prevalence was defined as any reported value (number; percentage) of DDH cases in Saudi Arabia, either at a one time-point or over time. We defined clinical practice for DDH as any form of clinical (conservative, rehabilitation, medical or surgical) intervention used to treat children with DDH in Saudi Arabia.

### Study Quality Assessment

Two independent authors (FK, MA) assessed the methodological quality of the included studies using the Study Quality Assessment Tools (SQAT; https://www.nhlbi.nih.gov/health-topics/study-quality-assessment-tools). The SQAT uses a text-based description to classify observational or experimental studies as follows: Good, Fair, or Poor based on specific questions related to internal validity [42]. Two authors (FK, MA) met and discussed results from the quality assessment and reached a final decision for each study. In the case that no agreement between the two authors (FK, MA) could be reach, a third author (NA) was consulted to reach a final decision on study quality assessment.

### Data Synthesis

In this final stage, we summarized and synthesized our findings using a thematic analysis for all included studies. Our measures included incidence (ratios) and prevalence (percentages) of DDH cases in Saudi Arabia, risk factors associated with DDH, and reported clinical practice interventions that were delivered in Saudi Arabia to treat those with DDH.

## Results

### Literature Search

**Fig. 1:**
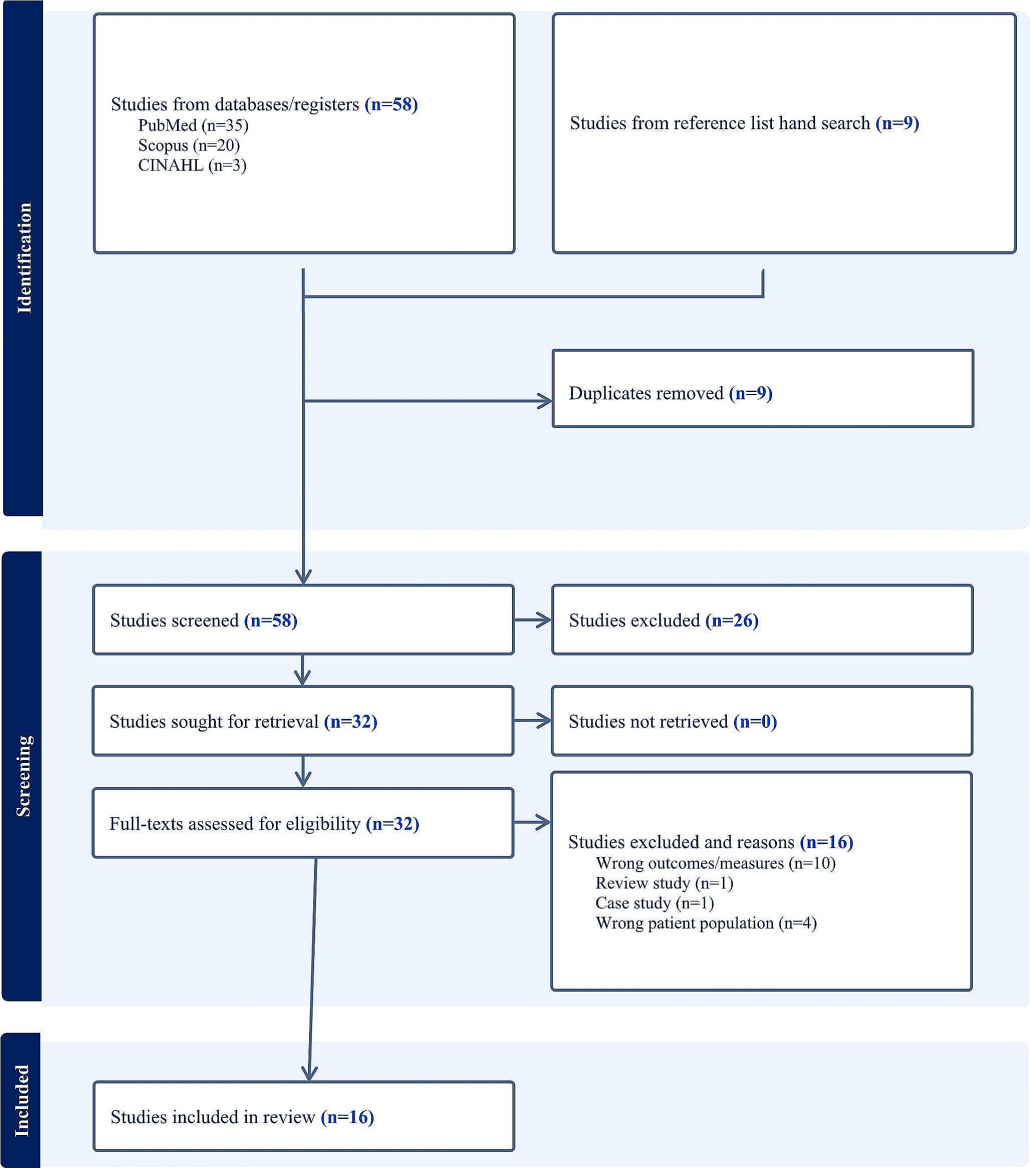
PRISMA flow chart

Our literature search yielded 67 potential studies (database searches, *n* = 58; manual/hand references searches, *n* = 9). Duplicates were removed (*n* = 9), and the remaining 58 studies were screened. Out of these 58 studies, 26 were excluded, and 32 studies were retrieved for full-text review. Of these 32 studies for which full texts were reviewed, 16 were excluded (wrong outcomes/measures, *n* = 10; wrong patient population, *n* = 4; review study, *n* = 1; and case study, *n* = 1), and the remaining 16 studies were included in this systematic review study [38, 39, 43–55], (Fig. 1). All the 16 included studies were available in English.

### Overall Description of all Included Studies in the Current Systematic Review

The age range of patients included in the studies in this systematic review ranged from 2.5 months to 86.4 months (combined total DDH sample: *n* = 3,127). In most of the included studies, bilateral DDH cases were more prevalent than unilateral DDH cases (Table 1). Several DDH diagnostic criteria/screening methods were reported, including the Ortolani and Barlow test [56], limited hip abduction range of motion, shortening of the thigh, impaired gait, hip instability test, patients’ medical files, history of open reduction with Pemberton or Dega acetabuloplasty, ultrasound radiographs, and/or computer tomography scans (Table 2). Studies were conducted in different regions of Saudi Arabia, including the Riyadh Region (central region, *n* = 11) [38, 39, 43–45, 47, 48, 51–53, 55], the Northern Region (*n* = 2) [50, 54], Aseer Region (southern region, *n* = 1) [30], the Almadinah Region (western region, *n* = 1) [46], and the Eastern Region (*n* = 1) [49]. Included studies were published between 1988 [49] and 2023 [38, 39, 43–48, 50–55].

**Table 2:** Summary table of demographic and diagnostic data, incidence, prevalence, and risk factors

Author (year)	Sample Size Overall (DDH)	Age (Months, M ± SD)	F:M Ratio	Involved Side (%: B/R/L)	Diagnosis Criteria	Incidence Rate (per 1,000 case)	Prevalence (%)	Associated Anomalies (%)	Risk Factors
Al-Umran et al. (1988) [49]	12,733 (62)	-	4.2:1	27.4/25.8/46.8	Ortolani and Barlow test	4.9	-	14.5	Female sex, breech presentation
Dargan (2001) [50]	439 (83)	-	1.8:1	43.0/26.0/31.0	-	-	18.9	-	Female sex
Mirdad (2002) [13]	79,548 (300)	14.5 ± 19.7	3.6:1	49.8/22.3/27.3	Ortolani and Barlow test, limited hip abduction, shortening of the thigh, gait disturbance. and X-ray	3.8	-	-	Female sex, positive family history, delivery with cephalic presentation
Kremli et al. (2003) [21]	(600)	7.6	6.0:1	36.3/26.5/37.1	-	-	-	6.2	Female sex, family history, delivery with cephalic presentation, breech position
Zamzam et al. (2009) [30]	(23)	6.2	3.6:1	100.0/0.0/0.0	Radiological diagnosis	-	-	-	Female sex
Alsiddiky et al. (2012) [51]	(88)	7.9 ± 1.6	5.3:1	40.9/10.2/48.9	Hip instability test	-	-	-	Female sex
Alassaf (2017) [52]	(128)	25.4 ± 8.1	-	-	Radiological diagnosis	-	-	-	-
Alanazi et al. (2017) [53]	955 (300)	7.5	220: 80	13.3/28.3/41.6	Clinical abduction Radiological diagnosis	3.1	-	-	Age, breech presentation
Jawadi et al. (2017) [54]	(574)	16.3	479: 95	45.0/ 25.4/29.6	Patients’ files	-	-	-	Family history, first born, breech presentation, and Oligohydramnios
Alassaf (2019) [43]	(50)	-	3.1:1	-	History of Dega or Pemberton acetabuloplasty	-	-	-	-
Alassaf (2020) [44]	(39)	-	1.7:1	-	Radiological diagnosis	-	-	-	-
Alsiddiky et al. (2020) [45]	(70)	20.8	7.8:1	100.0/0.0/0.0	History of open reduction with Pemberton or Dega acetabuloplasty	-	-	-	-
Ibrahim et el. (2020) [22]	(73)	-	3.1:1	57.5/15.1/27.4	Ultrasound, X-ray, and Computer Tomography	-	-	24.7	Female sex, family history, Cesarean section, age < 3 years
Rehab et al. (2020) [46]	50 (39)	2.5	2:1	0.0/41.0/59.0	Ultrasound	-	78	-	-
Vasilcova et al. (2022) [47]	12,225 (678)	86.4 ± 6.0	3.6:1	-	Radiograph or ultrasound		6.0	-	-
Bakarman et al. (2022) [48]	(20)	22.17 ± 5.9	6:1	0.0/42.1/57.9	History of Dega or Pemberton acetabuloplasty	-	-	-	-

F = Female, M = Male, N = Number, SD = Standard Deviation, DDH = Developmental Dysplasia of the Hip, R = Right, L = Left, B = Bilateral

Out of the 16 included studies, 3 studies reported incidence [30, 49, 54], another 3 studies [38, 39, 49] reported prevalence, 9 studies reported risk factors [38, 39, 49–52, 54, 55], and 10 studies reported clinical practice approaches used among children diagnosed with DDH in Saudi Arabia [43–45, 47–49, 51–54]. Four studies reported data related to early screening programs used to examine children with DDH in Saudi Arabia [49, 51, 52, 54]. Out of 16 included studies, 3 studies used cross-sectional study designs [39, 46, 54] and the remaining 13 studies used cohort study designs (retrospective, *n* = 9 [30, 43, 44, 47, 48, 50, 52, 53, 55]; prospective, *n* = 4 [38, 45, 49, 51]). The quality assessment of the included studies (Table 3) showed that 11 studies were of a high-quality rating (i.e., “good”) [39, 43–45, 47–49, 30, 52–54], whereas the remaining 5 studies were of a moderate-quality rating (i.e., “fair”) [38, 46, 50, 51, 55].

### Description of Incidence and Prevalence Rate of DDH Condition in Saudi Arabia

Out of 16 included studies, 3 studies reported the incidence rate of DDH in Saudi Arabia [30, 49, 54]. In 1988, the incidence rate of DDH in Saudi Arabia was estimated to be 4.9 cases per 1,000 births [49]. In 2017, another study reported that incidence of DDH in Saudi Arabia was 3.1 cases per 1,000 births [54]. Most recently, in 2022, the incidence rate of DDH in Saudi Arabia was estimated to be 3.8 cases per 1,000 births [30]. Table 2 describes characteristics of all included studies that reported incidence data.

Out of 16 included studies, 3 studies reported prevalence of DDH in Saudi Arabia [38, 39, 49]. In 2001, the prevalence of DDH in Saudi Arabia was estimated to be 18.9% [50]. In recent years, two studies that were conducted in 2020 [46] and 2022 [47] reported that the prevalence of DDH in Saudi Arabia was 78% and 6.0%, respectively (Table 2).

### Description of Risk Factors Associated with DDH Diagnosis in Children in Saudi Arabia

Out of 16 included studies, 9 studies reported risk factors associated with a DDH diagnosis in Saudi Arabia [38, 39, 49–52, 54, 55], including female sex [*n* = 7] [38, 39, 49–52], breech position [*n* = 4] [38, 49, 54, 55], family history [*n* = 4) [30, 38, 39, 55], delivery with cephalic presentation [*n* = 2] [30, 38], Oligohydramnios, birth by Cesarean section, and age of less than 3 years (*n* = 1) [55] (Table 2).

### Clinical Practice Approaches Used among Children with DDH in Saudi Arabia

Four studies out of the 16 included studies reported on the use of conservative treatments [30, 43, 47, 49], including closed reduction and brace application (e.g., Rosen, Pavlik, Tibungen, Frejka, Aberdeen, Coxaflex, or Teufel). Nine studies of the 16 included studies reported on the use of surgical treatment options [43–45, 48, 30–54], including open hip reduction, adductor tenotomy, capsulotomy, pelvic osteotomy, and/or acetabuloplasty (Dega or Pemberton) (Table 3).

### Follow-Up Data in Children Diagnosed with DDH in Saudi Arabia

Out of 16 included studies, 7 studies [44, 45, 48, 49, 51–53] reported follow-up data ranging from 3 and up to 49 months (Table 3). Adverse events have been observed in these 7 studies [44, 45, 48, 49, 51–53], including hip dislocation, avascular necrosis, interrupted shortened line, and/or migration percentage > 29%. Failure rate for surgical and conservative treatments has been documented in these 7 studies [44, 45, 48, 30–53], ranging from 5.6 to 21.6% (Table 4).

**Table 3:** Summary table of study characteristics and treatment provided

Author (year)	Design	Study Quality Assessment*	Region / City	Screening Duration (years)	Early Screening	Treatment Provided (type)	Specific Treatment
Al-Umran et al. (1988) [49]	Prospective Cohort study	Good	Eastern Region / Al-Khobar	5	Yes	Conservative (Rosen splint)	Rosen splint
Dargan (2001) [50]	Retrospective cohort study	Fair	Northern Borders / Rafha	8	No	-	-
Mirdad (2002) [13]	Retrospective cohort study	Good	Aseer / Abha	4	No	Surgical and conservative	-
Kremli et al. (2003) [21]	Prospective cohort study	Fair	Riyadh / Riyadh	5	No	-	-
Zamzam et al. (2009) [30]	Prospective cohort study	Fair	Riyadh / Riyadh	5	Yes	Surgical	Open reduction
Alsiddiky et al. (2012) [51]	Retrospective cohort study	Good	Riyadh / Riyadh	3	Yes	Surgical	K-wire fixation
Alassaf (2017) [52]	Retrospective cohort study	Good	Riyadh / Riyadh	4	No	Surgical	Open reduction
Alanazi et al. (2017) [53]	Cross-sectional study	Good	Northern Borders / Arar	2.11	Yes	Surgical and conservative	Open reduction and conservative
Jawadi et al. (2017) [54]	Retrospective cohort study	Fair	Riyadh / Riyadh	6.11	No	-	-
Alassaf (2019) [43]	Retrospective cohort study	Good	Riyadh / Riyadh	4	No	Conservative vs. surgical	Closed reduction or open reduction and pelvic osteotomy
Alassaf (2020) [44]	Retrospective cohort study	Good	Riyadh / Riyadh	3	No	Surgical	Dega or Pemberton acetabuloplasty
Alsiddiky et al. (2020) [45]	Prospective cohort study	Good	Riyadh / Riyadh	11	No	Surgical	Adductor tenotomy, capsulotomy, open reduction, and acetabuloplasty
Ibrahim et el. (2020) [22]	Cross-sectional study	Good	Riyadh / Riyadh	4	No	-	-
Rehab et al. (2020) [46]	Cross-sectional study	Fair	Al-Madinah Al-Madinah	0.3	No	-	-
Vasilcova et al. (2022) [47]	Retrospective cohort study	Good	Riyadh / Riyadh	5	No	Conservative	Pavlik, Tibungen, Frejka, Rosen, Aberdeen, Coxaflex, or Teufel brace
Bakarman et al. (2022) [48]	Retrospective cohort study	Good	Riyadh / Riyadh	5	No	Surgical	Dega or Pemberton acetabuloplasty

* Evidence quality assessed using the National Heart, Lung, and Blood Institute’s Study Quality Assessment Tools

**Table 4:** Summary table of follow-up data and adverse outcomes/events

Author (year)	Follow-up Data Reported	Follow-up Time Points (months)	Dislocated Hips After Follow-up, n	Avascular Necrosis (Hips), n	Interrupted Shortened Line, n	Migration Percentage > 29%, n	Failure Rate (%)
Al-Umran et al. (1988) [49]	Yes	3, 6, 12	1	-	-	-	-
Dargan (2001) [50]	No	-	-	-	-	-	-
Mirdad (2002) [13]	No	-	-	-	-	-	16.0
Kremli et al. (2003) [21]	No	-	-	-	-	-	-
Zamzam et al. (2009) [30]	Yes	3,6,9,12	-	3	-	-	13.0
Alsiddiky et al. (2012) [51]	Yes	25–56	3	4	-	-	5.6
Alassaf (2017) [52]	Yes	2–49	4	-	4	27	21.6
Alanazi et al. (2017) [53]	No	-	-	-	-	-	-
Jawadi et al. (2017) [54]	No	-	-	-	-	-	-
Alassaf (2019) [43]	No	-	-	-	-	-	-
Alassaf (2020) [44]	Yes	> 18	2	6	-	-	13.8
Alsiddiky et al. (2020) [45]	Yes	Every 6 weeks during the first 6 months, minimum of 24 months	2	2	-	-	5.7
Ibrahim et el. (2020) [22]	No	-	-	-	-	-	-
Rehab et al. (2020) [46]	No	-	-	-	-	-	-
Vasilcova et al. (2022) [47]	No	-	-	-	-	-	-
Bakarman et al. (2022) [48]	Yes	> 26	-	-	-	-	10.0

## Discussion

This systematic review is the first study, to our knowledge, that synthesized the current evidence regarding incidence, prevalence, risk factors, and clinical practice approaches for children with DDH in Saudi Arabia (16 studies were included). We found that the incidence rate of DDH in Saudi Arabia ranged from 3.1 to 4.9 cases per 1,000 births [30, 49, 54] and that the prevalence ranged from 6 to 78% [38, 39, 49]. We further found that female sex, family history of DDH, breech delivery, oligohydramnios, Cesarean section, and age less than 3 years old were the primary reported risk factors associated with DDH in those in Saudi Arabia. We also found that brace application was the most-reported conservative intervention, whereas the most-common surgical interventions included, open hip reduction, adductor tenotomy, capsulotomy, pelvic osteotomy, and/or acetabuloplasty. Lastly, the majority of the studies included DDH participants who lived in the central region of Saudi Arabia (i.e., Riyadh) [38, 39, 43–45, 47, 48, 51–53, 55], and majority of our included studies were of a good quality rating [39, 43–45, 47–49, 30, 52–54].

Compared to the incidence rate found in this systematic review (3.1 to 4.9 per 1,000 births), the incidence rates of DDH reported in other countries are higher. For example, in Canada, the incidence rate of DDH has been reported as approximately 6.6 cases per 1,000 births and 2.2 cases of late-detected DDH cases per 1,000 births [57]. In the United States, the incidence of DDH was reported as 11.5 per 1,000 live births per the American Academy of Pediatrics [58]. Furthermore, a recently-published systematic review that included 43 studies reported an average pooled incidence rate of DDH as 9.8 cases per 1,000 births, diagnosed using a universal ultrasonographic screening method [59]. Compared to the DDH prevalence found in the current systematic review (6% and up to 78%), the prevalence rates of DDH reported in other countries is lower. An Australian study reported that the prevalence of DDH was as low as 1.0% in 1991 [220 per 19,622 live births; year: 1991]; however, this study did not explicitly mention their diagnostic criteria used to estimate the prevalence of DDH cases [60]. Additionally, a Brazilian study (*n* = 678; year: 2021) reported that the prevalence of DDH among newborns in Brazil was 5.5% [61]. Another previous study in China (*n* = 25,767; year: 2017) reported that the prevalence rate of DDH was approximately 1.52% [62]. The incidence and prevalence of DDH in our current study as compared to those reported in other studies from other countries may vary based on several factors, such as the study location, sample size, and/or the DDH diagnostic criteria used. In addition, the inconsistencies observed in the incidence and prevalence from the included studies in the current systematic review could be due to several reasons. The higher incidence rates found in some countries could be due to well-established screening guidelines encouraging the use of ultrasound and/or radiographic imaging techniques [63–65]. Furthermore, in Saudi Arabia, there may be missed cases due to either the lack of sufficiently-trained professionals [66] or due to a lack in community awareness regarding signs and symptoms of DDH, primarily within families [67]. Furthermore, we observed in one of our included studies that the reported prevalence of DDH was particularly high (78%.) [46]. This extreme finding regarding prevalence of DDH can be attributed to the use of a highly-sensitive diagnostic instrument for detecting DDH cases [46], coupled with the absence of any subsequent follow-up procedures. Furthermore, a significant proportion of infants exhibiting minor hip articular abnormalities experience spontaneous resolution, [68] and this could potentially explain why the study may have overestimated the prevalence of DDH in Saudi Arabia.

There has been a growing trend in the utilization of universal ultrasonographic screening [59], an effective method for reducing the incidence of late-detected DDH. Using this screen technique enables clinicians to easily and more accurately examine potential DDH cases in early age in order to intervene earlier, when indicated, in an effort to improve outcomes and reduce disability in this patient population [59]. Regarding risk factors for DDH, we found that female sex was the most frequently reported risk factor associated with DDH development found in this systematic review [*n* = 7 studies] [38, 39, 49–52], followed by breech presentation (*n* = 4 studies) [38, 49, 54, 55]. The least frequently reported risk factor in this study was oligohydramnios (*n* = 1 study) [55]. Importantly, newborns with a family history of DDH, being born female, being aged less than 3 years, and exhibiting associated abnormalities were found to have an approximately 16, 3, 2.5, and 2 times increased risk for developing DDH, respectively [39]. Similarly, a study conducted in the United States reported risk factors associated with DDH, and these included female sex, family history of DDH, and breech position during birth [69]. Regarding diagnosis, the presence of risk factors along with positive clinical signs have a increase the likelihood of DDH [70]. Additionally, a previous study conducted in Japan reported that female sex was less important risk factor for DDH when compared to other risk factors like breech delivery and family history [71]. Indeed, in a study from Australia, breech presentation was the predominant risk factor identified for DDH cases [60]. Infants who are born with breech presentation through vaginal delivery may exhibit a greater susceptibility to developing DDH compared to those who were delivered via Caesarean section, with odds ratios of 25.6 and 9.5, respectively [60]. Our findings regarding risk factors of DDH condition are consistent with previously-reported findings across different studies in other countries, suggesting the importance of screening for common risk factors associates with DDH.

In this systematic review, we observed that 9 studies reported surgical intervention [43–45, 48, 30–54] and 4 studies reported conservative treatment [30, 43, 47, 49] used to treat DDH cases in Saudi Arabia. Previous research has reported that the course of treatment for children with DDH is contingent upon the child’s age and the reducibility of the hip joint [72]. During the initial stages of DDH identification, and until the age of 6 months, the suggested primary therapeutic approach involves the utilization of an abduction brace, such as Pavlik harness [73]. We further found that only one study recommended the use of surgical treatment during the first 6 months of age in those with DDH [51]. In this study, the main reason for using surgical intervention (open reduction) in early ages (i.e., before 6 months) was the failure of conservative treatment (e.g., Pavlik harness) [51]. Across all of the included studies, we observed that closed reduction was the first medical intervention recommended after the age of 6 months, followed by spica casting [43]. For older DDH patients (> 18 months), the typical course of recommended treatment involves surgical intervention; mainly open reduction and/or hip reconstruction surgery [74]. Our findings regarding DDH treatment course among children in Saudi Arabia are in line with other clinical practice guidelines in other countries [75]. Lastly, regarding treatment failure among children with DDH in Saudi Arabia, we observed that the reported failure rates in Saudi Arabia were comparable to failure rates that were reported in other studies/countries (Saudi Arabia: ranges from 5.6 to 21.6%; other studies: ~18%) [76, 77].

The current systematic review could help to guide clinicians in Saudi Arabia working with newborns and children who are at high-risk of developing DDH, using the synthesized data reported in this study. Future studies should examine the longitudinal impact of DDH on disability as well as overall health, to assist in the initiation of tailored conservative and/or surgical interventions in this patient population. Furthermore, further studies should focus on determining the incidence and prevalence of DDH in Saudi Arabia to guide clinicians and researchers through providing epidemiological statistics. Establishing DDH registry in Saudi Arabia could also help in providing information that aid screening and treatment of this condition. The current systematic review has several limitations that need to be considered when interpreting its findings. Using meta-analysis was not possible in this study, due to the lack of evidence on the topic and heterogeneity of data reported across the included studies. Some of our included studies used different techniques to either diagnose or treat DDH condition in Saudi Arabia [39, 43, 52–54, 44–49, 30, 51]. However, we did perform a through, qualitative synthesize of the data to provide an overall summary of DDH incidence, prevalence, risk factors, and treatment options for patients in studies conducted in Saudi Arabia. Additionally, other relevant studies that evaluated any of the outcomes of interest might have been missed during the search. However, we used a comprehensive search strategy that was reviewed several times to ensure all relevant key terms were used in order to retrieve all potential studies.

## Conclusion

In this systematic review, we found that the incidence and prevalence of DDH in Saudi Arabia is relatively differ compared to other countries. The lower incidence and higher prevalence of DDH cases in Saudi Arabia may be attributed to the lack of established protocols for early detection of DDH, insufficient awareness among healthcare practitioners, and/or inconsistent utilization of standardized screening methods such as physical examination, radiography, and ultrasound within high-risk patients. Commonly-reported risk factors included female sex and breech birth position. Brace application was commonly used to treating children with DDH before the age of 6 months, and surgical intervention was preferred for those of older ages (> 18 months) or if a previous conservative intervention failed.

## Electronic Supplementary Material

Below is the link to the electronic supplementary material.

Supplementary Material 1

Supplementary Material 2

## Data Availability

No datasets were generated or analysed during the current study.

## Abbreviations

DDH: Developmental dysplasia of the hip
PRISMA: Preferred Reporting Items for Systematic Reviews and Meta-Analyses
SQAT: Study Quality Assessment Tools
