# Correction: Incidence, Prevalence, Risk Factors, and Clinical Treatment for Children with Developmental Dysplasia of the Hip in Saudi Arabia. A Systematic Review

**DOI:** 10.1007/s44197-024-00224-6

**Published:** 2024-04-15

**Authors:** Naif Alrashdi, Mansour Alotaibi, Moqfa Alharthi, Faizan Kashoo, Sultan Alanazi, Ahmad Alanazi, Msaad Alzhrani, Thamer Alhussainan, Rami Alanazi, Rakan Almutairi, Matthew Ithurburn

**Affiliations:** 1 Department of Physical Therapy and Health Rehabilitation, College of Applied Medical Sciences, Majmaah University, AL-Majmaah, 11952 Saudi Arabia; 2 Department of Physical Therapy, College of Applied Medical Sciences, Northern Border University, Arar, Saudi Arabia; 3 Rehabilitation Services Department, King Abdullah Specialized Children’s Hospital, Ministry of National Guard Health Affairs, Riyadh, Saudi Arabia; 4 Department of Orthopedics, King Faisal Specialist Hospital & Research Center, Riyadh, Saudi Arabia; 5 Department of Physical Therapy and Rehabilitation, King Khaled Hospital, Almajmaah, Saudi Arabia; 6 Physiotherapy Department, Al Iman General Hospital, Riyadh First Health Cluster, Riyadh, Saudi Arabia; 7 American Sports Medicine Institute, Birmingham, AL USA; 8 Department of Physical Therapy, School of Health Professions, University of Alabama at Birmingham, Birmingham, AL USA

**Correction: Journal of Epidemiology and Global Health**

10.1007/s44197-024-00217-5

After this article was published, the author informed us that the name of the 7th author was mistyped. It appeared as “Alzahrani”, while it should be correctly “Alzhrani”

Also, in the first paragraph of the Methods section a blockade for the Prospero number was erroneously not solved. It should have been “CRD42023433646” instead of “XXX”.

The Original article has been corrected.

