# Supplementary material for: Incidence, Prevalence, Risk Factors, and Clinical Treatment for Children with Developmental Dysplasia of the Hip in Saudi Arabia. A Systematic Review
Source: J Epidemiol Glob Health. 2024 Mar 14;14(3):549–60. doi: 10.1007/s44197-024-00217-5 (PMC11444034; doi:10.1007/s44197-024-00217-5)
Supplement: Supplementary file 1 — Supplementary Material 1 [file 44197_2024_217_MOESM1_ESM.pdf]

www.asmi.org
American Sports Medicine Institute

November 15, 2023

Dear Editorial Board of *the Journal of Epidemiology and Global Health*:

Please see our attached manuscript entitled, "Incidence, Prevalence, Risk Factors, and Clinical Treatment for Children with Developmental Dysplasia of the Hip in Saudi Arabia. A Systematic Review."

We believe this manuscript will be of interest to the readership of the journal and look forward to your and the reviewers' comments. Additionally, this manuscript has not been submitted elsewhere for publication.

Please do not hesitate to contact me should you require more information.

Sincerely,

Matt Ithurburn, PT, DPT, PhD
Director of Clinical Research
American Sports Medicine Institute
205.918.2126 | matti@asmi.org
