# Supplementary material for: Incidence, Prevalence, Risk Factors, and Clinical Treatment for Children with Developmental Dysplasia of the Hip in Saudi Arabia. A Systematic Review
Source: J Epidemiol Glob Health. 2024 Mar 14;14(3):549–60. doi: 10.1007/s44197-024-00217-5 (PMC11444034; doi:10.1007/s44197-024-00217-5)
Supplement: Supplementary file 2 — Supplementary Material 2 [file 44197_2024_217_MOESM2_ESM.docx]

**Title Page:**

**Title:** Incidence, Prevalence, Risk Factors, and Clinical Treatment for Children with Developmental Dysplasia of the Hip in Saudi Arabia. A Systematic Review

Naif Z. Alrashdi, PT, MSRS, PhD;^1^ Mansour M. Alotaibi, PT, PhD, MBA;^2^ Moqfa S. Alharthi, PT, MSc;^3^ Faizan Z. Kasho, PT, MSc;^1^ Sultan A. Alanazi, PT, MSc;^1^ Ahmad D. Alanazi, PT, MSc, PhD;^1^ Msaad M. Alzahrani, PT, MSc, PhD;^1^ Thamer Alhussainan, MD;^4^ Rami Alanazi, PT;^1,5^ Rakan Almutairi, PT;^1,6^ and Matthew P. Ithurburn, PT, DPT, PhD^7,8^

1. Department of Physical Therapy and Health Rehabilitation, College of Applied Medical Sciences, Majmaah University, AL-Majmaah, 11952, Saudi Arabia
2. Department of Physical Therapy, College of Applied Medical Sciences, Northern Border University, Arar, Saudi Arabia.
3. Rehabilitation Services Department, King Abdullah Specialized Children's Hospital, Ministry of National Guard Health Affairs, Riyadh, Saudi Arabia
4. Department of Orthopedics, King Faisal Specialist Hospital & Research Center, Riyadh, Saudi Arabia
5. Department of Physical Therapy and Rehabilitation, King Khaled Hospital, Almajmaah, Saudi Arabia
6. Physiotherapy Department, Al Iman General Hospital, Riyadh First Health Cluster, Riyadh, Saudi Arabia
7. American Sports Medicine Institute, Birmingham, Alabama, United States
8. Department of Physical Therapy, School of Health Professions, University of Alabama at Birmingham, Birmingham, Alabama, United States

**Key Words:** disability, early screening, acetabular dysplasia, DDH, infants, Saudi Arabia

**Corresponding author:** Naif Z. Alrashdi, PT, MSRS, PhD, Department of Physical Therapy and Health Rehabilitation, Majmaah University, Al-Majmaah, 11952, Saudi Arabia; [n.alrashedy@mu.edu.sa](mailto:n.alrashedy@mu.edu.sa); Phone number: 00966559035192

**Funding:** King Salman Center for Disability Research (Award ID: KSRG-2023-414)

**Acknowledgement:** The authors extend their appreciation to the King Salman center For Disability Research for funding this work through Research Group no KSRG-2023-414

**Conflict of interests:** We have no conflicts of interest to declare related to this work
